# Hypocalcemia in Covid-19: A Prognostic Marker for Severe Disease

**DOI:** 10.30699/IJP.2020.130491.2442

**Published:** 2020-10-24

**Authors:** Ahmad Raesi, Ebrahim Saedi Dezaki, Hamideh Moosapour, Farzane Saeidifard, Zahra Habibi, Fereidoun Rahmani, Soleiman Kheiri, Elham Taheri

**Affiliations:** 1 *Department of Internal Medicine, Clinical Research Development Unit, Hajar Hospital, Shahrekord University of Medical Sciences, Shahrekord, Iran*; 2 *Department of Parasitology, School of Medicine, Shahrekord University of Medical Sciences, Shahrekord, Iran*; 3 *Evidence Based Medicine Research Center, Endocrinology and Metabolism Clinical Sciences Institute, Tehran University of Medical Sciences, Tehran, Iran*; 4 *Department of Medicine, Northwell Health-Lenox Hill Hospital, Zucker School of Medicine at Hofstra/Northwell, New York, United States of America (USA)*; 5 *Division of Preventive Cardiology, Department of Cardiovascular Disease, Mayo Clinic, Rochester, United States of America (USA)*; 6 *Department of Infectious Disease, Clinical Research Development Unit, Hajar Hospital, Shahrekord University of Medical Sciences, Shahrekord, Iran*; 7 *Department of Epidemiology and Biostatistics, School of Health, Modeling in Health Research Center, Shahrekord University of Medical Sciences, Shahrekord, Iran*; 8 *Molecular Pathology and Cytogenetic Ward, Pathology Department, School of Medicine, Shiraz University of Medical Sciences, Shiraz, Iran*

**Keywords:** Albumin, COVID-19, Clinical severity, Electrolyte -imbalances, Hypocalcemia, Parathyroid hormone, Prognosis, Vitamin D

## Abstract

**Background & Objective:**

Previous studies have addressed the electrolyte abnormalities such as hypocalcemia in COVID-19 patients. We aimed to compare the laboratory findings especially the electrolyte levels among COVID-19 patients and healthy controls and evaluate their prognostic values.

**Methods:**

This case-control study included 91 COVID-19 patients and 169 healthy individuals. Their laboratory parameters including electrolytes, albumin, liver enzymes, complete blood count, vitamin D, and parathyroid hormone (PTH) were compared. We also analyzed the association between these markers and the major outcomes including severity, mortality and hospitalization.

**Results:**

Among patients with COVID-19, 59.3% of the patients had hypocalcemia on admission while in control group only 32.5% had low calcium level (OR=3.02, 95% CI: 1.79-5.13, *P*<0.001). The rates of death and ICU admission were significantly higher among the patients in hypocalcemic group than those of eucalcemic group (85.7% vs 14.3% and 33.3% Vs 9.1%, respectively). However, there was no significant difference in the mean PTH and vitamin D levels between the two groups. In terms of the severity of the infection, 74.1% of patients in hypocalcemic group had a severe infection while 24.3% of the patients in eucalcemic group were diagnosed with severe infection (OR=8.89, 95% CI: 3.38-23.37, *P*<0.001).

**Conclusion:**

Patients with COVID-19 may present with considerable laboratory abnormalities including hypocalcemia. The hypocalcemia would be also associated with worse major clinical outcome and higher mortality risk.

## Introduction

Pneumonia caused by COVID-19 infection is an emerging infectious disorder that has gained a worldwide public health attention (1). The world health organization (WHO) has issued diagnostic strategies and treatment recommendations and suggested classifying COVID-19 disease into 4 stages: mild, moderate, severe and critical (2). Recent studies have reported the clinical characteristics and outcomes of COVID-19 with different severity (3-5). The underlying mechanisms of the novel coronavirus leading to disease progression and organs dysfunction remain to be explored. Due to the high mortality and the lack of effective treatments in critical patients (6-8), early identification of these patients is crucial. If we know the risk factors for progression of the patients to severe or critical stages of COVID-19, we can identify the groups with most likely poor outcomes, so that, we can focus on prevention and treatment efforts. Recent studies have reported that patients admitted into the intensive care unit (ICU) had more abnormal serum parameters (7). While lymphopenia, elevated lactate dehydrogenase, liver enzymes, creatinine kinase (CK) and C-reactive protein (CRP) are among the most common laboratory findings of COVID-19 patients, other laboratory parameters such as electrolyte imbalances are also increasingly have been recognized as important factor among COVID-19 patients (4,6,7). The electrolyte imbalance, especially hypocalcemia, plays an important role in the patients' health management (1), and may be involved in the pathophysiologic mechanisms dictated by this viral agent. We observed a high incidence of hypocalcemia in the COVID-19 patients with severe disease. Therefore, we hypothesized that serum calcium levels were associated with the disease severity and prognosis of patients with COVID-19 infection. 

Accordingly, we designed a case-control study to investigate the electrolyte imbalances; especially hypocalcemia, in the patients affected by COVID-19, and compared findings to a matched control group with no COVID-19 infection. Also, we aimed to investigate the association between the level of calcium and major clinical outcomes including mortality, ICU admission and duration of hospitalization in the COVID-19 patients.

## Materials and Methods

**Patients and Controls**

This case-control study was carried out on adults ≥18 years of age who referred to Hajar Hospital, Shahrekord University of Medical Science, Shahrekord, Iran, with COVID-19 infection from February to April 2020 with their matched healthy controls. The clinical outcomes such as mortality, organ injury, and discharges were monitored until the time of their discharge or death until April 26, 2020. 

Our cases included the patients with definitive COVID-19 disease confirmed by a positive real-time reverse transcriptase-polymerase chain reaction (RT-PCR) assay on the nasopharyngeal swab specimens (9). Hypocalcemia was defined as a serum calcium level less than 8.6 mg/dL.

The severity of the COVID-19 infection was classified based on the WHO-China joint mission. Mild: minor clinical symptoms (e.g., fever and cough) with no manifestations on imaging; Moderate: fever, or respiratory tract infection symptoms with imaging indicating pneumonia; Severe: meet at least one of the following criteria: respiratory rate≥30 breaths/min, oxygen saturation (SpO_2_)≤93%, lung infiltration >50% of the lungs on imaging, and require ICU admission (2).

A control group of 169 healthy adults were selected using the data registry of a reference laboratory in the same city. The age, sex and laboratory findings (including electrolytes, albumin, liver enzymes, complete blood count, vitamin D, and parathyroid hormone (PTH)) of the control group were retrieved from the laboratory database and matched with the cases.

These control individuals were healthy persons without any serious medical disorders that referred to the laboratory from December 1 to January 20, 2019 prior to the pandemic of COVID-19 by their primary care physicians for the routine checkup. We did not select control individuals from the hospitalized patients during the pandemic to avoid any risk of false negative or false positive results for the COVID-19 infection. 

**Data Collection**

Data including age, sex, initial symptoms and duration of hospitalization was collected from the electronic medical records. The serum levels of calcium, aspartate aminotransferase (AST), alanine aminotransferase (ALT), alkaline phosphatase (ALP), albumin, blood urea nitrogen (BUN), creatinine, sodium, potassium, white blood cell (WBC) count, lymphocyte count, hemoglobin, vitamin D, PTH and O_2_ saturation were recorded within 24 hours of admission. 

**Ethical Considerations**

Although most information was extracted from data available on clinical records of individuals, data on some laboratory tests (e.g., parathyroid hormone, vitamin D) were required for the research purposes while these tests had not been performed for the clinical purposes. These tests were performed by the use of serum samples of the patients which routinely stored and then left over after diagnostic procedures. Consequently, it was not required to repeat venipuncture and blood collection, thus, patients were not bothered to take additional risk or harm for the research purposes. Therefore, the implicit consent was obtained from the patients during their clinical workups. Furthermore, to protect the confidentiality and privacy of individuals, strategies such as coding were employed during data registry and analysis.

**Statistical Analysis**

The data were summarized as frequencies and percentages for the categorical variables and Means ± SD for the continuous ones. Differences between variables among groups were analyzed using independent sample T-test and Chi-square test where appropriate. Logistic regression was used to calculate odds ratios (OR) with 95% confidence intervals for the categorical outcomes. The analysis was performed by SPSS version 23 and STATA version 0.05 and statistical significance was defined as P-value<0.05.STATA version 0.05 was used to create the Figures.

## Results

All 91 PCR-confirmed COVID-19 patients and 169 controls enrolled in the study were older than 18. The age ranged from 18 to 90 years old. Cough and dyspnea were prominent in 83.3% and 73.8% of patients, respectively which were recorded as the main initial symptoms. Duration of hospitalization was from 1 to 15 days with a mean of 7.32 ± 3.03 days. The rate of ICU admission was 24.4%. Fourteen patients with severe COVID-19 infection (16.3%) were expired. Other characteristics of the patients are shown in Table 1. The range, mean ± SD, and the percentage of abnormal values for some admission laboratory findings of 91 COVID-19 patients are shown in Table 2.

**Table 1:** Clinical Characteristics of 91 Patients with COVID-19

Parameter	Mean ± SD/ %	Range	Abnormal (%)
Pre-admission signs and symptoms			
Respiratory rate	21.76± 6	14-57	43.5%>20
Heart rate	86.17±19.2	37-148	7.5%<60 &11.2% >100
Diastolic blood pressure	74.21 ± 9.3	50-90	3.9%<60
Systolic blood pressure	117.57 ± 14.2	70-150	1.3%<90 & 2.3%>140
Oral temperature	37.92 ± 0.7	36-39.7	81.6 >37.3
O2 saturation	86.06 ± 11 11.24	40-97	50
Cough	83.3%		
Dyspnea	73.8%		
Pre-admission co-morbidities			
Hypertension	27.2		
Diabetes mellitus	16.2		
Ischemic Heart Disease	12.5		
Hyperlipidemia	11.4		
COPD ^*^	8.6		
Hypothyroidism	4.9		
Asthma	2.4		
Chronic kidney disease	0		
Chronic liver disease	0		
Outcome			
Duration of hospitalization	7.46 ± 3.03	1-15	
ICU admission	24.1		
Death	16.3		
			

^*^ Chronic Obstructive Pulmonary Disease

**Table 2:** Admission Laboratory Findings of 91 COVID-19 Patients

Parameter	Mean ± SD/ %	Range	Abnormal (%)
Pre-admission signs and symptoms			
Respiratory rate	21.76± 6	14-57	43.5%>20
Heart rate	86.17±19.2	37-148	7.5%<60 &11.2% >100
Diastolic blood pressure	74.21 ± 9.3	50-90	3.9%<60
Systolic blood pressure	117.57 ± 14.2	70-150	1.3%<90 & 2.3%>140
Oral temperature	37.92 ± 0.7	36-39.7	81.6 >37.3
O2 saturation	86.06 ± 11 11.24	40-97	50
Cough	83.3%		
Dyspnea	73.8%		
Pre-admission comorbidities			
Hypertension	27.2		
Diabetes mellitus	16.2		
Ischemic Heart Disease	12.5		
Hyperlipidemia	11.4		
COPD ^*^	8.6		
Hypothyroidism	4.9		
Asthma	2.4		
Chronic kidney disease	0		
Chronic liver disease	0		
Outcome			
Duration of hospitalization	7.46 ± 3.03	1-15	
ICU admission	24.1		
Death	16.3		
			

^*^Statistical Significance at P-value<0.05

Comparing the cases with the controls, there was a significant difference in the number of patients with hypocalcemia (corrected serum calcium <8.6). The percentages of hypocalcemia were 59.3% and 32.5% in the cases and control groups, respectively (OR=3.02, 95% CI: 1.79-5.13, *P*<0.001). Moreover, the mean serum calcium level of COVID-19 patients at the time of admission was significantly less than this level in control group (8.36 ± 0.57 vs 8.79 ± 0.35 mg/dL, *P*< 0.001). However, differences in the vitamin D and PTH levels did not reach the statistical significance. Also, there were significant differences in the mean levels of other laboratory findings in the patients affected by COVID-19 compared to the healthy controls which are presented in Table 3. .

**Table 3:** Admission Laboratory Findings of COVID-19 Patients in Comparison with Those of Controls

	COVID-19 (n=91)	Controls (n=169)	P-value
Variable	Mean ± SD or %	Mean ± SD or %	
Sex (male)	60.4%	66.9%	0.34
Age	55.44 ± 16.9	55.91 ± 15.2	0.82
Calcium (mg/dL)^ †^	8.36 ± 0.57	8.79 ± 0.35	< 0.001^*^
Hypocalcemia^†† ^^	59.3%	32.5%	<0.001^*^
Vitamin D (nmol/L)	73.16 ± 23.59	76.02 ± 23.48	0.37
PTH (pg/mL)	45.13 ± 23.2	46.25 ± 10.82	0.545
White Blood Cell (×10 ^9^/μL)	7.330 ± 4.5441	6.636 ± 1.83	0.16
Lymphocyte (×10 ^9^/μL)	1.163 ± 5.88	3.49 ± 0.89	< 0.001^*^
Hemoglobin (g/dL)	14.0 ± 2	14.5 ± 1.9	0.05^*^
Albumin (g/dL)	3.73 ± 0.49	4.52 ± 0.2	< 0.001^*^
BUN (mg/dL)	16.45 ± 10.33	16.45 ± 4.7	0.176
Creatinine (mg/dL)	0.84 ± 0.27	0.92 ± 0.16	0.002^*^
Sodium (mmol/L)	136.97 ± 3.34	141.6 ± 1.3	< 0.001^*^
Potassium (mmol/L)	4.08 ± 0.37	4.46 ± 0.57	< 0.001^*^
ALT (U/L)	32.32 ± 41.92	24.3 ± 12.36	0.084
AST (U/L)	33.93 ± 53.92	18.44 ± 6.1	0.008^*^
ALK (U/L)	153.46±39.01	201.98 ± 42.23	< 0.001^*^

* Statistical Significance at P-value<0.05

† Calcium levels were corrected by albumin (corrected calcium=calcium + (0.8 × (4-albumin)))

†† Hypocalcemia means serum calcium level ≤8.6 mg/dL

^ OR=3.02, 95% CI: 1.79-5.13

In evaluation among the COVID-19 patients, the association between serum calcium level and their clinical outcome was investigated by two types of analysis: calcium level measurement and degree of severity of the disease.

Firstly, patients were divided into two groups (as shown in Table 4) based on their serum calcium values. The rates of death and ICU admission were significantly higher among the patients in hypocalcemic group than those of eucalcemic group (85.7% vs 14.3% and 33.3% Vs 9.1%, respectively). However, there was no significant difference in the mean PTH and vitamin D levels between the two groups. In terms of the severity of the infection, 74.1% of patients in hypocalcemic group had a severe infection while 24.3% in eucalcemic group were diagnosed with severe infection (OR=8.89, 95% CI: 3.38-23.37, *P*<0.001). The rate of hypocalcemia was not significantly different between male and female patients (65% vs 77%, respectively; *P*=0.34).

**Table 4:** **Comparison of **clinical and laboratory findings in 91 COVID-19 patients in regard to the calcium level

Variable	Hypocalcemic patients ^†^ (n=54)	Eucalcemic patients ^††^ (n=37)	P-value
	Mean ± SD / Percentage	Mean ± SD / Percentage	
Sex (male)	60 %	40 %	0.52
Age (year)	55.67 ± 15.9	55.11 ± 18.4	0.88
Hospitalization (day)	7.96 ± 3.1	6.66 ± 2.8	0.17
ICU admission	33.3%	9.1%	0.008^*^
Death	22.06%	6.1%	0.04^*^
Severity	81.6%	18.4%	<0.001^*^
Dyspnea	61.3%	38.7%	0.37
O2 saturation	84.26 ± 12.35	89.07 ± 7.57	0.05^*^
WBC (×10^9^/μL)	7.94 ± 5.24	6.43 ± 3.11	0.9
Lymphocyte (×10^9^/μL)	1.09 ± 0.56	1.25 ± 0.61 0.5	0.2
Hemoglobin (g/dL) male	14.9 ± 1.5	14.7 ±1.75	0.65
Hemoglobin (g/dL) female	12.79 ± 2.1	12.68 ± 1.35	0.86
Albumin (g/dL)	3.75 ± 0.54	3.7 ± 0.42 0.3	0.68
Vitamin D (nmol/L)	73.89 ± 20.38	72.1 ± 19.22	0.73
PTH (pg/mL)	46.79 ± 26.75	42.57 ±16.46	0.41

† Serum calcium level ≤8.6 mg/dL

†† Serum calcium level >8.6 mg/dL and ≤ 10.5

* Statistical significance at *P*< 0.05

Secondly, patients were divided into categories of severity based on the above-mentioned criteria. While there was no patient with mild infection, 53.8% were classified as severe and the rest as moderate infection. Table 5 compares the clinical and laboratory findings between the patients with moderate and severeCOVID-19 infection. The rate of severe disease in male patients was not significantly different from the females (55.5% vs 52.7%, respectively; *P*=0.83)

The serum calcium levels showed significant positive and negative correlations with O_2_ saturation and respiratory rates, respectively. These results were independent of the vitamin D and PTH levels in COVID-19 patients (as the levels were not significantly different between the cases and controls as well as patients with moderate and severe diseases).

Regarding the age factor, the mean age of cases and controls were 55.44±16.9 and 55.91±15.2, respectively; as the controls were matched according to the age, as expected, no significant difference was found (*P*=0.82, as shown in Table 3). Also, the mean age of hypocalcemic and eucalcemic patients was not significantly different (55.67 vs 55.11, respectively, *P*=0.87). Furthermore, the level of calcium was not correlated with the age factor (Pearson=-0.08 and *P*=0.44).

Regarding sex, 60.4% cases and 66.9% controls were male; as the controls were matched according to the sex, as expected, no significant difference was found (*P*=0.34, as shown in Table 3). Among the COVID-19patients, 54. 2% of males and 52.8% of females had severe diseases (*P*=0.5). Additionally, the rate of death as final outcome in the female patients was higher, but the difference was not statistically significant (24.2% vs 11.3%, respectively and *P*=0.14). And finally, the mean of calcium level in male and female patients was not significantly different (8.34 vs 8.38, respectively and *P*=0.79).

**Fig. 1:**
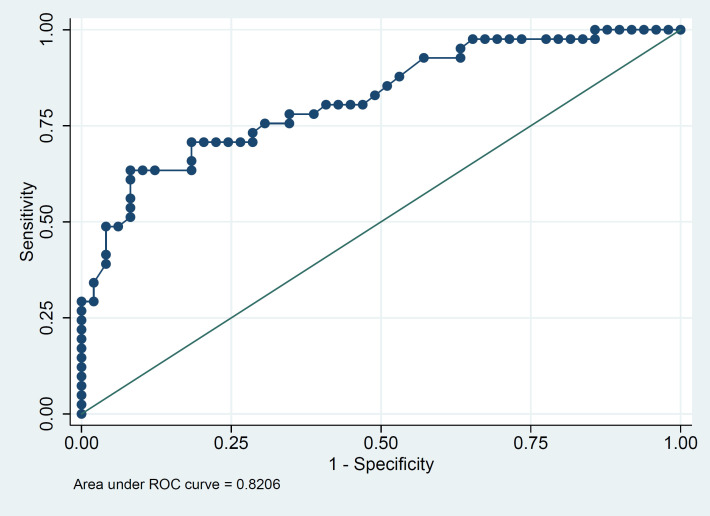
ROC curves showing the association between serum calcium level and severity of disease. The optimal cut-off point for the severity was 8.69mg/dL; sensitivity 91.8%, and specificity 63.4%

**Fig. 2:**
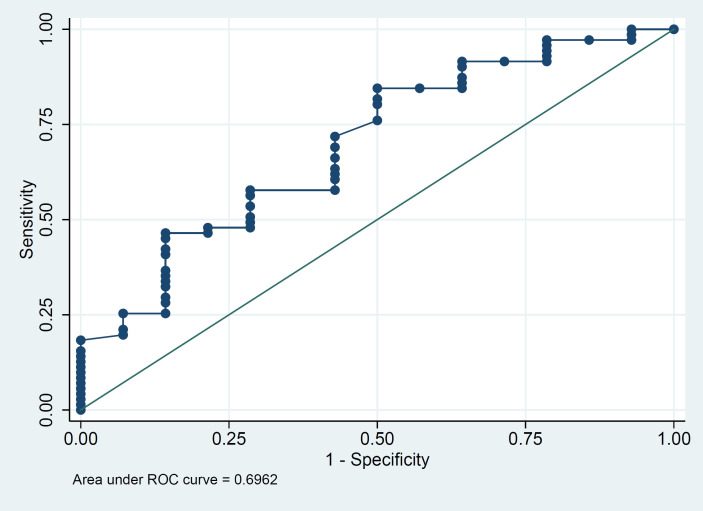
ROC curves showing the association between the serum calcium level and mortality. The optimal cut-off point for the mortality was 8.38 mg/dL; sensitivity 71.4%, and specificity 57.7%).

The ROCs were also calculated to assess the associations between serum calcium level and severity of the infection as well as mortality (Figures 1 and 2). The optimal cut-off points of serum calcium values were derived from the ROCs. The optimal cut-off point for the severity was 8.69 mg/dL, (sensitivity 91.8%, and specificity 63.4%). The optimal cut-off point for mortality was 8.38 mg/dL, (sensitivity 71.4%, and specificity 57.7%).

**Table 5:** Comparison of clinical and laboratory findings in 91 COVID-19 patients in regard to the clinical severity

	Moderate (n=42)	Severe (n=49)	P-value
Variable	Mean ± SD / N (%)	Mean ± SD / N (%)	
Sex (male)	59.5	61. 2	0.51
Age (year)	49.6 ± 15.4	60.39 ± 16.7	0.02^*^
Hospitalization(day)	6.66 ± 2.8	7.96 ± 3.09	0.05
ICU admission	0	42.9	<0.001
Death	0	29.2	<0.001
Respiratory rate	20 ± 4.03	22.82 ± 6.66	0.03
O2 saturation	89.07 ± 5.02	84.26 ± 12.35	<0.001^*^
WBC(×10^9^/μL)	6.74 ± 3.48	7.83 ± 5.26	0.25
Lymphocyte(×10^9^/μL)	1.32 ± 0.55	1.02 ± 0.58	0.01^*^
Hemoglobin(g/dL) male	15 ± 1.6	14.6 ± 1.5	0.31
Hemoglobin(g/dL) female	12.7±1.2	12.7±2.2	0.8
Albumin(g/dL)	3.88 ± 0.47	3.60 ± 0.48	0.007^*^
BUN(mg/dL)	14 ± 6.89	18.55 ± 12.24	0.029
Creatinine(mg/dL) male	0.82 ± 0.15	1 ± 0.43	0.05
Creatinine(mg/dL) female	0.71 ±0.12	0.94±0.53	0.08
Sodium(mmol/L)	137.5 ± 2.8	136.4 ± 3.6	0.11
Potassium(mmol/L)	4.11± 0.38	4.05 ± 0.37	0.42
Vitamin D(nmol/L)	70.7 ± 19.2	75.2 ± 26.8	0.40
PTH(pg/mL)	43.08 ± 17.2 ± 16.44	47.0 ± 27.68 ± 28.01	0.47
Calcium (mg/dL)	8.69 ± 0.41	8.08 ± 0.54	< 0.001^*^

^*^Statistical significance at P-value< 0.05

## Discussion

This study demonstrated the association between total serum calcium level and the severity of the infection as well as the major outcome in the patients with COVID-19 infection. This is one of the first studies that addressed this issue in COVID-19 patients and healthy control group. Although nowadays there are suggestive methods for the diagnosis and treatment of COVID-19 disease, the mortality rate of critical patients with severe disease is still high which is important to identify the risk factors for the severe illness or death (10). However, few reports have been published to establish an early and sensitive biomarker to predict the severity of the disease and prognosis of COVID-19, especially in Iran. In this study, we found that serum calcium level would be associated with the severity of the disease and prognosis of the patients with COVID-19.

Mild hypocalcemia has already been shown to be common in the patients with SARS and Ebola infections (11,12). Since the beginning of the pandemic, a few studies have addressed hypocalcemia as an important laboratory finding in COVID-19 patients (13-15). The prevalence of hypocalcemia was 59.3% in our patients. This result was in line with the previous published studies. In the studies conducted by Sun *et al.*, and Cao* et al.*, 74.7% and 65.4% of the COVID-19 patients, respectively had hypocalcemia (13, 14). 

One of our key findings was that patients with lower level of serum calcium (especially ≤ 8.6mg/dL) exhibited a more severe form of COVID-19 infection and a higher rate of ICU admission and mortality. These findings are consistent with the results of the study by Sun *et al.* (13).

Comparing COVID-19 patients regarding sex and age and the correlation between these factors and calcium levels or clinical outcome showed that age and sex were not confounding variables.

Calcium homeostasis is regulated by the vitamin D and the PTH-calcium axis. Dysfunction of PTH is highly prevalent in the critical illnesses (16), and hypocalcemia is common in critically ill patients (17). Moreover, hypocalcemia is associated with the organ dysfunction and poor outcomes in critically ill patients (18). Interestingly, hypocalcemia in our patients was independent of vitamin D and PTH levels, and these measures were not significantly different between our cases and controls. However, Sun *et al.*, revealed that hypocalcemia was associated with hypoproteinemia, imbalanced vitamin D and PTH levels in the acute phase of COVID-19 infection (13). Hence, our observation provides a new insight into the correlation between hypocalcemia and COVID-19 infection. It is essential to identify the mechanism of action of the virus, as well as the risk factors that lead to more severe illness or death (19). The underlying pathophysiology of the multi-organ dysfunction due to COVID-19 infection is still unknown. It is also unknown if there is a higher risk of hypocalcemia associated with COVID-19 or if COVID-19 infection causes hypocalcemia. Several studies have indicated that the concentration of inflammatory markers including the cytokines is higher in the serum of the COVID-19 patients than in healthy adults (20, 21). Pro-inflammatory cytokines such as interleukin-1 (IL-1) and interleukin-6 (IL-6) are important mediators of the acute response to the critical illness. The association between hypocalcemia and more severe infection in our study can be justified by the interplay between serum calcium level and the immune system that has been proposed by others (22-25). The findings of this study which were consistent with the previous reports, confirmed that serum calcium level would be associated with the severity of the disease and prognosis of the patients with COVID-19.

Additionally, our study revealed that patients with COVID-19 infection had lower sodium and potassium level compared to the control group and appropriate management of these imbalances will be useful in the treatment of COVID-19 patients. Li *et al.*, showed that hypokalemia is known to exacerbate acute respiratory distress syndrome (ARDS) and acute cardiac injury, which are considered as common complications in COVID-19 patients (26).

This study faced some limitations that need to be discussed. We had a small sample size that could affect the results, but most of the results were in line with the previous studies that indicates the reliability of our study. We also measured the total calcium serum level rather than ionized calcium, which may not precisely reflect the exact total level of the calcium.

## Conclusion

This study highlighted the high incidence of hypocalcemia in the patients with severe COVID-19 infection. Patients with lower total serum calcium levels (especially ≤ 8.45 mg/dL) had worse clinical outcome, and higher mortality rate. The serum calcium level could be used as a prognostic marker for the mortality rate.

## Data Availability

The data sets during and/or analyzed during the current study available from the corresponding author on reasonable request.
